# TERMINOLOGY USED BY EMPLOYERS AND JOB SEEKERS FOR AGING-RELATED POSITIONS

**DOI:** 10.1093/geroni/igz038.187

**Published:** 2019-11-08

**Authors:** Leanne J Clark-Shirley, Tina Kruger Newsham, M A Guest

**Affiliations:** 1 AARP, Washington, District of Columbia, United States; 2 Indiana State University, Terre Haute, Indiana, United States; 3 University of Kentucky, Lexington, Kentucky, United States

## Abstract

Our aging society calls for a workforce capable of meeting older adults’ diverse needs. Yet the extent that employers seek out a workforce with aging-related training or education is unclear, as is how people with such backgrounds search for positions. We describe an exploratory content analysis of job postings to understand how employers are searching for applicants with aging-related backgrounds, and compare job posting keywords to terms used by a sample of aging-trained job seekers/employees. Results showed 35% of aging-related job postings used keywords expressing preference for applicants with aging-related backgrounds; the most commonly-occurring terms were “gerontology,” “Assisted living” + “adult day” + “director” + “nursing home administrator,” and “elderly.” Job seekers also cited “gerontology” as a term used to search for positions, along with “aging,” “older adults” and “seniors”. Findings suggest that employers should use more positively-connoted terms to attract applicants with aging-related backgrounds, rather than terms like “elderly.”

